# EURL ECVAM Genotoxicity and Carcinogenicity Database of Substances Eliciting Negative Results in the Ames Test: Construction of the Database

**DOI:** 10.1016/j.mrgentox.2020.503199

**Published:** 2020

**Authors:** Federica Madia, David Kirkland, Takeshi Morita, Paul White, David Asturiol, Raffaella Corvi

**Affiliations:** a European Commission Joint Research Centre, Ispra, Italy; b Kirkland Consulting, Tadcaster, UK; c Chemical Management Center, National Institute of Technology and Evaluation (NITE), Nishihara, Shibuya-ku, Tokyo, Japan; d Environmental Health Science and Research Bureau, Health Canada, Ottawa, CA

**Keywords:** Carc, carcinogenicity, CAvit, in vitro chromosomal aberration test, CAviv, in vivo chromosomal aberration test, CCRIS, Chemical Carcinogenesis Research Information System, CGX, Carcinogenicity and Genotoxicity eXperience, CPDB, Carcinogenic Potency Database, CSCL, Japanese Chemical Substances Control Law, CTA, cell transformation assay, DNAviv, in vivo comet and alkaline elution assays, ECHA, European Chemicals Agency, EFSA, European Food Safety Authority, EURL ECVAM, EU Reference Laboratory for Alternatives to Animal Testing, GENE-TOX, Genetic Toxicology Data Bank, Hprt, hypoxanthine-guanine phosphoribosyl transferase locus, HSDB, Hazardous Substances Data Bank, HESI GTTC, Health and Environmental Sciences Institute’s (HESI) Genetic Toxicology Technical Committee, IARC, International Agency for Research on Cancer, ISHL, Japanese Industrial Safety and Health Law, JETOC, Japan Chemical Industry Ecology-Toxicology and Information Center, MCGM, mammalian cell gene mutation, MLA, mouse lymphoma Tk gene mutation assay, MNvit, in vitro micronucleus test, MNviv, in vivo micronucleus test, NTP, National Toxicology Program, OECD, Organisation for Economic Cooperation and Development, OECD SIDS, OECD Screening information data set, OFG, Organic functional group, QSAR, Quantitative Structural Activity Relationship, SCE, sister chromatid exchange, SCCS, Scientific Committee on Consumer Safety, TGR, Transgenic rodent gene mutation assay, UDSviv, in vivo unscheduled DNA synthesis test, Negative Ames tests, genotoxicity, carcinogenicity, database, chemical space, EURL ECVAM

## Abstract

The bacterial reverse mutation test (Ames test) is the most commonly used genotoxicity test; it is a primary component of the chemical safety assessment data required by regulatory agencies worldwide. Within the current accepted *in vitro* genotoxicity test battery, it is considered capable of revealing DNA reactivity, and identifying substances that can produce gene mutations via different mechanisms. The previously published consolidated EURL ECVAM Genotoxicity and Carcinogenicity Database, which includes substances that elicited a positive response in the Ames test, constitutes a collection of data that serves as a reference for a number of regulatory activities in the area of genotoxicity testing. Consequently, we considered it important to expand the database to include substances that fail to elicit a positive response in the Ames test, i.e., Ames negative substances. Here, we describe a curated collection of 211 Ames negative substances, with a summary of complementary data available for other genotoxicity endpoints *in vitro* and *in vivo*, plus available carcinogenicity data. A descriptive analysis of the data is presented. This includes a representation of the chemical space formed by the Ames-negative database with respect to other substances (e.g. REACH registered substances, approved drugs, pesticides, etc.) and a description of the organic functional groups found in the database. We also provide some suggestions on further analyses that could be made.

## Highlights

• EURL ECVAM Consolidated Genotoxicity and Carcinogenicity Database extended.
• Negative Ames test results were compiled and reviewed.
• A database of Ames negative results was constructed.
• Database chemical space characterization was conducted.
• OFG representation of carcinogens and non-carcinogens was characterised.

## 1 Introduction

The assessment of genotoxicity is an essential component of the safety assessment of all types of chemical substances (e.g., therapeutic products, pesticides, industrial chemicals, etc.); its requirement specified in national legislation (e.g., EU, Canada, USA, Japan) aims at the protection of human health. Genotoxicants are capable of inducing the genomic alterations that have been mechanistically and empirically linked to cancer and germ cell effects, the latter leading to fertility problems or heritable genetic disorders [1,2].

The assessment is performed in a step-wise fashion whereby a battery of *in vitro* tests in both bacterial and mammalian cells are employed to detect a variety of effects (e.g., mutations, structural and numerical chromosomal aberrations); in certain cases *in vitro* testing is followed by *in vivo* studies. The exact combination of *in vitro* and *in vivo* studies may vary depending on the outcomes of initial testing, and/or the type of substance under investigation, and/or the requirements of the relevant legislation; the strategies employed generally depend on jurisdictions [3,4].

In this context, within the current accepted *in vitro* genotoxicity test battery, the bacterial reverse mutation test (i.e., Ames test) is a testing cornerstone, generally representing the first step of the genotoxicity assessment [5]. It is capable of identifying substances that can elicit mutations (e.g., base-pair substitutions, frameshifts, insertions, deletions) via different mechanisms including, for example, secondary DNA damage due to generation of oxidative stress (e.g., reactive oxygen species). A positive response generally leads to the presumption that the chemical has the potential to be a carcinogen. The Ames test is employed worldwide as an initial screen to determine the mutagenic potential of new and legacy chemicals, as well as a wide range of substances (e.g., therapeutic products, pesticides, industrial chemicals, food additives) in the early stages of product development [6]. Thus, evaluating its predictivity for *in vivo* genotoxicity and carcinogenicity, when considered alone or in association with mammalian cell assays for chromosome damage and/or gene mutations, is essential.

To scrutinise the overall utility of the Ames test, the correspondence between Ames test results and *in vitro* mammalian cell genotoxicity test, and the ability of *in vitro* test combinations to predict *in vivo* genotoxicity and carcinogenicity, we previously published a consolidated EURL ECVAM Genotoxicity and Carcinogenicity Database of Ames-positive substances [[7], [8], [9]]. The curated database includes 726 Ames-positive substances with genotoxicity and carcinogenicity data obtained from different sources, e.g. publications, databases. The Ames-positive database is populated with all available information for genotoxicity endpoints *in vitro* and *in vivo*, plus available carcinogenicity data. It constitutes a powerful resource for developing and evaluating alternative approaches to animal testing for genotoxicity and carcinogenicity assessment and has proved its utility as reference source for a number of genotoxicity-related regulatory and research activities [10,11].

To further address the utility of the Ames test for the prediction of *in vivo* genotoxicity and carcinogenicity activity, or lack thereof, we have complemented the Ames-positive database with a database of substances that failed to elicit a positive response in the Ames test. This resulted in a curated collection of data for 211 Ames-negative substances. The database also includes available information for other genotoxicity endpoints *in vitro* and *in vivo* (e.g. chromosomal aberrations, micronucleus), plus carcinogenicity data.

Here, we present the strategy used for the construction of the Ames-negative database including an expert review of the data. The work also comprises a characterisation of the database composition in terms of chemical space covered, and relative prevalence of organic functional groups.

## 2 Construction of the database

The Ames-negative substances were identified as described below (Section 2.1); information was subsequently recorded in a master database with a structure analogous to that employed for the previously-constructed EURL ECVAM Genotoxicity and Carcinogenicity Database of Ames-positive test results [9]. In compiling the database the following rules were applied:

• Since the objective of the exercise is to construct a database that can ultimately be employed to evaluate the ability of the Ames test to predict presence or absence of *in vivo* genotoxic or carcinogenic activity, substances for which there were no valid carcinogenicity and no valid *in vivo* genotoxicity data - (CAviv), micronuclei (MNviv), UDS (UDSviv), transgenic mutations in rodents (TGR), or DNA damage (DNAviv) - were excluded.
• Entries of the database for free-bases and their corresponding salts, or for different salts of the same ion, were combined into single entries since it is expected that free-bases and their salts, for example, would behave identically in the aqueous environments pertaining to the *in vitro* and *in vivo* assays under consideration. This was the case for 11 substances: Aniline/Aniline hydrochloride; Ephedrine sulphate/Ephedrine hydrochloride; Erythromycin/Erythromycin stearate; Fluvastatin/Fluvastatin sodium salt; Fluphenazine/ Fluphenazine hydrochloride; Nitrilotriacetic acid/Nitrilotriacetic acid, trisodium salt, monohydrate; Saccharin/sodium Saccharin; Tetrakis (hydroxymethyl) phosphonium chloride/Tetrakis (hydroxymethyl) phosphonium sulfate; *m*-Toluidine/Toluidine hydrochloride; Toremifene/Toremifene citrate; Zinc sulfate/heptahydrateracemic form. In the case of Menthol, the D- and L-isomers were combined into one single entry as similar results had been obtained on testing the isomers separately.
• Substances such as complex hydrocarbon mixtures, gasoline fractions, paraffins, etc. for which the chemical composition of the test article was not defined and/or the identity and/or purity of the tested substance was not indicated, were excluded.
• Natural extracts and nanoparticles, namely Comfrey (*Symphytum officinale L.),* titanium dioxide and C60 nanoparticles, were also excluded from the database. In the first case, it was difficult to know whether the same extract was used across the different assessments. In the case of the latter, in several studies giving discordant results, the tested extracts were isolated either from dried milled roots or from leaves, and contained uncharacterised concentrations of the active pyrrolizidine alkaloid symphitine. Titanium oxide and C60 nanoparticles were excluded based on the recent work from the Health and Environmental Sciences Institute’s Genetic Toxicology Technical Committee (HESI-GTTC), which indicated that the Ames test is not recommended for genotoxicity assessment of nanomaterials [12].

### 2.1 Data sources and data collection

For a substance to be classified as negative in the Ames test, it was important to confirm that all strains required under OECD Test Guideline TG 471 [11] had been tested both in the absence and presence of metabolic activation (see Section 2.2.1). Therefore we started by collecting data from the Carcinogenicity and Genotoxicity eXperience (CGX) database of rodent carcinogens [4,13]. This provided an initial list of 500 potentially Ames-negative substances, which were then filtered using on-line resources to check that the Ames test results included all strains specified in OECD Test Guideline TG 471 [14]. More specifically, the various data sources used to construct the CGX were scrutinized according to the following iterative procedure:

• NTP source was scrutinized first [15]. The call present in the NTP source was used for the interpretation of the strain-specific results. If more results were available all were recorded. It appeared that the majority of the tests included the 4 basic *Salmonella* strains indicated in OECD TG 471 [14], i.e. TA98, TA100, TA1535 and either TA1537, TA97 or TA97a, but *S. typhimurium* TA102 and *E. coli* WP2 *uvrA* strains were missing. For this reason, the NTP studies that did not include *E. coli* or TA102 were considered alongside data from other sources, i.e. if data from *E. coli* or TA102 were reported in other databases and/or peer-reviewed publications, then together with the NTP data all relevant strains had been tested.
• In a second step, TOXNET [16] including a subset of databases (i.e., CCRIS, HSDB, GENE-TOX and CPDB) were analyzed. Most of these online databases are “secondary sources” since they only report dichotomous results (i.e., pos/neg) that were originally presented in other published documents. In most of the cases, the retrieval of the original sources for detailed analysis and verification was very difficult due to the fact they were very old (pre-1970).
• Finally, a free text search was used to query TOXLINE, PubMed and Google. For this search the common name of the compound was used instead of the CAS number. When some bacterial strains were missing (e.g. *S. typhimurium* TA102 or *E. coli* WP2 *uvrA*), the search parameters included the strain name such that only documents reporting that strain were included. If data for a specific compound were entirely absent, the search was more generic. Such searches often retrieved only documents containing an overall call for the Ames test, but no strain-specific information. This activity permitted retrieval of general reviews of existing data conducted by international organizations, and/or data contained in regulatory dossiers and databases such as:
  ○ EFSA Draft Assessment Reports; ECHA dossiers; European Union Risk Assessment Reports;
  ○ Japanese Chemical Substances Control Law and the Industrial Safety and Health Law Documents (i.e., CSCL-ISHL);
  ○ Japan Chemical Industry Ecology-Toxicology and Information Center (i.e., JETOC);
  ○ National Toxicology Program Technical Reports;
  ○ Reports published by the California EPA Department of Pesticide Regulation;
  ○ Screening Information Data Set (SIDS) published by OECD;
  ○ Publications related to the Joint FAO/WHO Meeting on Pesticide Residues (JMPR);
  ○ International Agency for Research on Cancer (IARC) Monographs.

These sources were subsequently queried using the CAS number. A similar approach was used to obtain missing test data for carcinogenicity and other *in vitro* and *in vivo* endpoints, or to resolve differences among different sources reporting results for the same substances. Particular attention was given to information presented in regulatory dossiers and peer-reviewed databases.

### 2.2 Data review

For each substance it was necessary to initially confirm the overall lack of activity in the Ames test, therefore, substances that were inconclusive, equivocal or positive were excluded from the database.

#### 2.2.1 Review of Ames data

Data were first collected and checked for compliance with the OECD Bacterial Reverse Mutation Test Guideline 471 [14]. Substances were considered “Ames negative” when results for all of the required strains, as described in OECD 471, showed negative results, both in the presence and absence of an exogenous source of metabolic activation [17].

As specified in the OECD 471, the recommended combination of strains is the following:

1. *S. typhimurium TA1535*, and
2. *S. typhimurium TA1537 or TA97 or TA97a*, and
3. *S. typhimurium TA98*, and
4. *S. typhimurium TA100*, and
5. *E. coli* WP2 *uvrA, or E. coli* WP2 *uvrA (pKM101), or S. typhimurium TA102*

Data from other bacterial strains not mentioned in the OECD 471 (e.g. TA104) were not considered.

It is worth mentioning that in a recent publication, Williams et al, reporting on the analysis performed by the International Workshop on Genotoxicity Testing (IWGT) group, have shown that few mutagens would fail to be detected if the test battery does not include Salmonella strains TA1535, TA1537, TA102, and *E. coli* WP2 *uvrA* [18].

Nonetheless, substances were considered Ames “inconclusive”, and excluded from the database, when results were negative across 1 or more publications or databases, but the full complement of strains had not been used, or some strains had not been tested with and without metabolic activation. The rigorous approach applied here is congruent with the recommendations of the OECD TG 471 as currently in place; it readily permits comparisons with Ames results collected previously.

#### 2.2.2 Review of data for other *in vitro* and *in vivo* genotoxicity endpoints and carcinogenicity

For each Ames-negative compound, results for the following tests were considered:

• *In vitro* genotoxicity: mouse lymphoma *Tk^+/−^* mutation assay and *Hprt* mutation assay (MCGM), micronucleus test (MNvit) and chromosome aberration test (CAvit). Although, most *in vitro* mutagenicity results pertained to the mouse lymphoma *Tk^+/−^* assay, for simplification the mammalian cell *Tk^+/−^* and *Hprt* mutation results were combined into a single category (i.e., MCGM). For simplicity, the category included also mammalian gene mutation in human TK6 cells.
• *In vivo* genotoxicity: MNviv, CAviv, TGR, unscheduled DNA synthesis (UDSviv), Cometviv (DNA damage including comet and alkaline elution). The TGR entries were primarily based on the results of transgenic rodent gene mutation assays, although for three substances, *Hprt* mutation *in vivo* and dominant lethal assay were also included in the TGR category.
• Carcinogenicity: rodent carcinogenicity.

Test results for all the assays listed above were curated via careful, expert review.

Information from other non-standard tests, including sister chromatid exchange in mammalian cells (SCEvit), DNA adducts *in vitro/vivo*, *in vitro* unscheduled DNA synthesis (UDSvit), cell transformation *in vitro* (CTA), or BlueScreen™ HC and GreenScreen® HC high-throughput gene reporter assays; were noted but not considered further. Data from tests performed in lower/other eukaryotes (e.g. *S. cerevisiae*, *D. melanogaster*, various plants) were not considered.

#### 2.2.3 Criteria for "overall calls"

In some cases, for the same substance, inconsistent results have been reported across publications and/or databases. For this reason, it was necessary to define an “overall call” for each *in vitro* and/or *in vivo* assay for each substance, especially in cases where results were inconsistent. For simplicity, the overall calls included only 4 categories, namely positive [+] or weak [+], negative [-], equivocal [E] or inconclusive [I].

Certain rules had to be applied to arrive at these overall calls; these are described below. In addition, a number of practical considerations were taken to confirm the test results, and define the overall calls. The quality of the study, the robustness of the protocol, and the perceived quality of the data (where available) were taken into account. For example:

1. Negative results from a recent Good Laboratory Practices (GLP) study conducted according to the most recent guidelines were considered more valuable than a negative result from an old study that did not comply with current guidelines.

2. Where conflicting results were reported in the different sources, the numbers of [+] and [-] calls were not considered as important as the quality and robustness of the tests, and whether the results had been obtained in different studies or from different publications, i.e., results from independent studies were considered more important than the same study result reported in different publications or databases (e.g., results from National Toxicology Program [NTP], [15] summarised in different publications).

Overall calls for each substance-endpoint combination were determined using the following criteria:

**An overall positive [+]** call for the *in vitro* studies was assigned regardless of whether the positive finding was noted only in the absence or only in the presence of S9, and of the cell type. A positive call was assigned irrespective of S9 source (i.e., rat, hamster or mouse).

**An overall positive [+]** call for the *in vivo* genotoxicity endpoints was assigned if there was clear evidence of a positive response from a single study (i.e., rats or mice, males or females). If there was clear evidence from more than one study, or if a substance was positive in one species or sex and negative in the other, a positive call was recorded. In the case of the latter, if two studies yielded different results, but it was clear that systemic exposures were greater in the positive than in the negative study, an overall call of [+] was given.

**An overall negative [-]** call for both *in vitro* and *in vivo* studies was assigned when all the requirements of the current OECD Test Guidelines or recommended best practices were fulfilled, and there was no evidence of a positive or equivocal response. A negative call was made for *in vivo* genotoxicity studies only if there was some direct or indirect evidence that the test substance reached the target tissue, otherwise it was considered inconclusive (see below). In instances where best practices had not been followed, but systemic toxicity could be inferred through read-across (e.g. observation of tumours in liver), an overall call of [-] was also assigned.

**An overall equivocal [E] call** was assigned if results were ambiguous, doubtful, questionable, or inconsistent (e.g., a positive and a negative test) within a study, or if a dose-related increase in effects was noted close to the borderline of biological significance, but the responses were not biologically and/or statistically significant, and no independent repeat experiment was done to verify the response. An [E] call was assigned in cases where there was some evidence of a positive response that could not be dismissed, but there were no consistent responses in the same test system across different studies. An [E] call was also assigned in cases where there were both positive and negative findings across different studies of apparently equal validity, and where the weight of evidence did not allow a clear positive or negative overall outcome.

**An overall inconclusive [I] call** was assigned in cases where no firm conclusion could be made in terms of meeting the requirements of the current OECD Test Guidelines or recommended best practices, e.g., negative studies only without S9 or only with S9; abnormal pH levels or osmolality in mammalian cell experiments; no evidence that adequate levels of toxicity were achieved; no proof of target cell exposure *in vivo*, only short treatment duration for the TGR assay, etc.. Overall [I] calls were also assigned if a positive response was noted in mammalian cells only above 10 mM or at high levels of cytotoxicity (i.e., close to or above recommended upper limit), and the available data did not permit a conclusion as to whether that chemical would elicit a positive response at acceptable levels of toxicity. Thus, an [I] call was assigned when the available information indicated that the result was not obtained via adequate testing, i.e., “no valid data”.

It should be noted that for the mammalian cell gene mutation (MCGM) assays, a negative result from a *Tk^+/−^*locus assay in mouse lymphoma cells was considered more robust than a negative result from an *Hprt* locus assay, which was usually performed in other cell types. The former detects both clastogenic and point mutations, whereas the latter predominantly detects point mutations. Thus, if positive results were reported in a *Tk^+/−^*assay alongside negative results from an *Hprt* assay, the positive *Tk^+/−^*results took precedence. If the only negative results were from the *Hprt* assay, this was noted alongside the overall call in the database. Since the NTP mouse lymphoma *Tk^+/−^*results have all been re-evaluated by Schisler et al., 2018 [19], according to current criteria [20], the re-evaluation calls were taken as being the most relevant, i.e., they would overrule the original NTP calls where different. Previously published calls of “Uninterpretable” (as used by [19]) were considered as Inconclusive [I].

Overall calls were assigned for carcinogenicity as follows:

**A positive [+] call** was assigned if a positive response was reported in either sex of rats or mice.

**A negative [-] call** was assigned if the substance was tested in both sexes of rats and mice, and all 4 groups yielded negative responses.

**An equivocal [E] call** was assigned if the substance was tested in both sexes of rats and mice, and there was some evidence of increased tumour frequencies, and at least one equivocal call was reported, and the other calls were negative.

**An inadequate, and therefore inconclusive [I], call** was assigned if the substance was tested in both sexes of rats and mice, and the results in at least one of the groups was considered to be compromised because of inadequate dosing (e.g., too low), excess mortality, or concurrent infection, and the other groups produced either negative or equivocal responses. If a substance was tested only in rats or mice, or only in one sex, and was not carcinogenic, that result was also considered inconclusive.

As in any evaluation, equivocal [E] responses form a unique category. It can be argued that, because the response is not clearly negative, [E] calls should be included in the positive response category based on precautionary principle. Thus, for the comparison of Ames-negative results to carcinogenic activity discussed below, [E] calls for carcinogenicity were considered as potentially positive alongside clear [+] or weak [+] calls.

## 3 Composition of the database

**Fig. 1:**
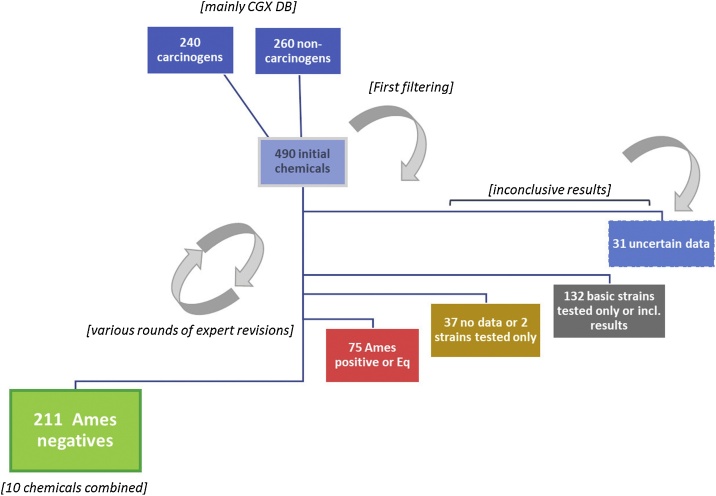
**Data review process for Ames test results.** Starting mainly from the CGX database, Ames test results from 500 substances were reviewed using a step-wise approach. After an initial screening, results for 490 chemicals were collated. Through several rounds of expert reviews, the following numbers of substances were removed: 69 substances that were found to have some positive or equivocal results, 37 substances with no data or tested only in two strains (i.e., mainly TA98 and TA100), 132 substances tested in a limited number of *S. typhimurium* strains (e.g., TA98, TA100, TA1535, TA1537 or TA97) or with inconclusive results, and 31 substances for which it was not possible to verify data sources and details. From the remainder, the combination of free-bases and their salts, different salts, or isomers (10 chemicals combined in total) resulted in a final collection of 211 Ames-negative substances. The rounds of expert review also involved scrutiny of *in vitro* and *in vivo* genototoxicity test results and carcinogenicity test results.

The database is presented in full as an online supplementary electronic file (Supplementary Table 1). Starting from an initial collection of 500 substances, mainly taken from the aforementioned CGX database, the various data review stages (described below in Fig. 1) provided a final database of 211 substances with clear Ames-negative results, but also with some *in vivo* genotoxicity or carcinogenicity data, representing 222 unique CAS numbers when the salts and isomers (see above) are considered separately (Supplementary Table 1). The remaining substances were either inconclusive, due to the lack of data for the full complement of standard strains or low data quality; detailed review in some cases resulted into positive or equivocal calls. The latter cases will be added in a revised version of the Ames positives EURL ECVAM consolidated Genotoxicity and Carcinogenicity database [9].

**Fig. 2:**
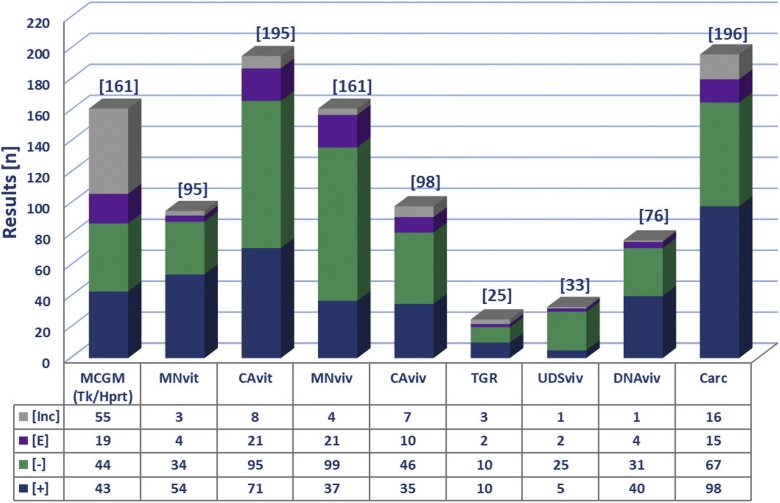
**Test results distribution.** Distribution of test results (i.e., overall calls) for the 211 Ames-negative substances. Data were collected for the following tests: *in vitro* mammalian cell gene mutation tests [MCGM], which includes mouse lymphoma *Tk^+/−^* or *Hprt* studies, *in vitro* micronucleus tests [MNvit], *in vitro* chromosome aberration tests [CAvit], *in vivo* tests including MN, CA, UDS, TGR, DNA damage [DNAviv] (i.e., Comet and alkaline elution assay,) and rodent carcinogenicity [Carc]. The total number of results for each test are reported in brackets.

In total, the database includes > 6000 entries and 1515 references covering the various *in vitro, in vivo* genetoxicity and carcinogenicity test results (Supplementary Table 1). Using the aforementioned criteria, analysis of all relevant database entries was used to assign overall calls for each test substance-endpoint combination. A summary of the database composition and the distribution of overall calls across all of the other *in vitro* and *in vivo* tests and the carcinogenicity studies is shown in Fig. 2. It is interesting to note that overall positive and negative results are scattered fairly consistently across the different tests. This is in contrast with the Ames-positive substances database where the overwhelming majority of responses in other test systems and endpoints were positive [8].

### 3.1 Categories of test article types

**Fig. 3:**
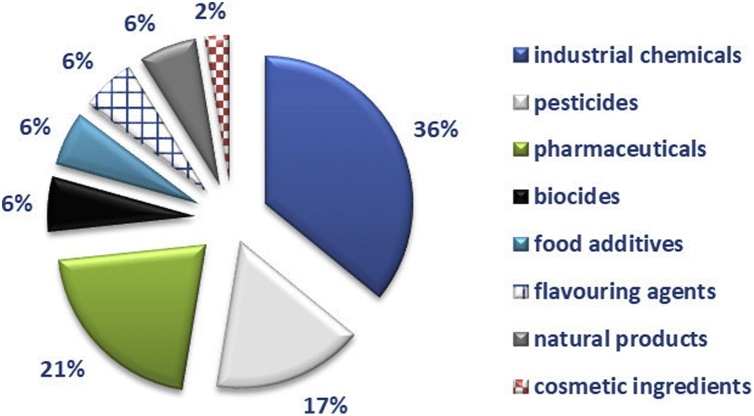
**Product type categories within the Ames-negative database.** Substances were categorised based on their main use and manufacturing as reported in PubChem and ChemIDplus databases.

Since the Ames-negative substances were scattered across different product sectors, we conducted an overall evaluation of their main use and manufacturing using the following sources: *PubChem* database, https://pubchem.ncbi.nlm.nih.gov/ and ChemID*plus* database, https://chem.nlm.nih.gov/chemidplus/. The results of this evaluation are shown in Fig. 3. The majority of substances in the database are industrial substances, followed by pharmaceutical products and pesticides. The database also includes a number of food additives, biocides, flavouring agents, natural products and cosmetic ingredients. It needs to be noted though, that this is a non-exhaustive analysis, and that some substances may have multiple uses.

### 3.2 Physicochemical properties

**Fig. 4:**
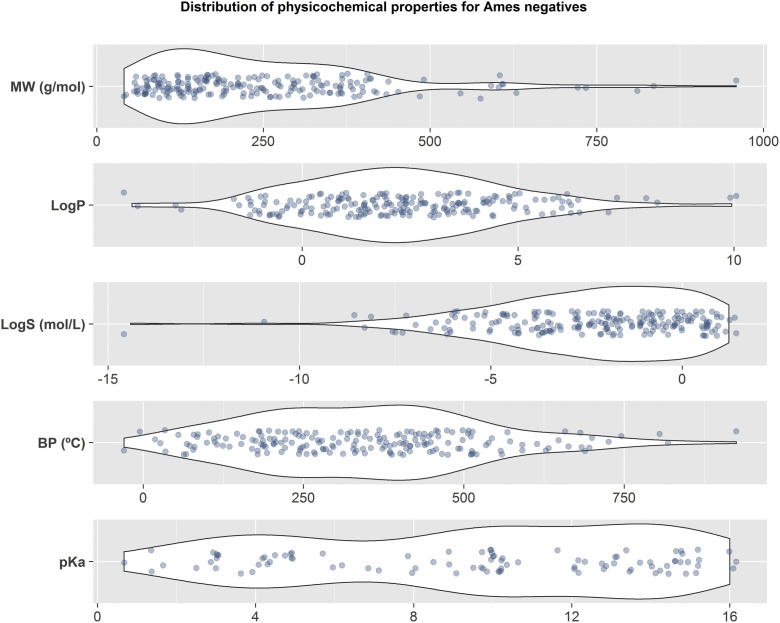
**Physicochemical properties of Ames-negative database.** The physicochemical properties of Ames-negative substances were calculated using Percepta/ACDLabs [21]. From top to bottom they correspond to: molecular weight (MW, g/mol), octanol-water partition coefficient (LogP), water solubility at pH 7.0 (LogS, mol/L), boiling point (BP, °C), and strongest acid pKa (pKa). The figure shows the values for each substance as dots, and the violin plots represent the number of substances with similar values along the axes. The violins are wider in the regions with more substances (i.e., higher density). The reported values correspond to those calculated for the desalted neutral substances that have a defined structure and are not metals or metallic salts (198 substances in total).

The main physicochemical properties of the substances in the Ames-negative database are shown in Fig. 4 as superposed *violin* and dot plots. In particular, these plots show the properties of each of the substances as well as their distribution. The dots represent the values for each of the properties of the substances, and the *violin* shapes indicate the number of substances with such values (density). The properties represented in Fig. 4 include molecular weight (MW in g/mol), octanol-water partition coefficient (logP), solubility at pH = 7.0 (LogS in mol/L), boiling point (BP in °C), and the lowest pKa. These physicochemical properties were calculated using the software Percepta/ACDLabs [21]. As is general practice, the properties were calculated on the desalted and neutral forms of the substances. In general, predictive software cannot predict the properties of metals, salts, or charged species. For the same reason no values could be obtained for the metallic salts and substances without a defined structure: Fosetyl Al, Gallium arsenide, Gum arabic, Lead acetate, Mercuric chloride, Nickel (II) sulfate hexahydrate, SD-0203, SD-2007, Sodium arsenite, Sodium fluoride, Tetrakis(hydroxyethyl)phosphonium chloride, Vanadium pentoxide, and Zinc sulfate. Therefore, the figure only includes values for 198 substances out of the 211 that comprise the database.

The fact that the distributions of all properties have Gaussian-like shapes (see Fig. 4), indicates that the database contains substances with typical physicochemical properties and that the database is not biased towards certain types of chemicals with very specific properties. It can be observed in Fig. 4 that most of the substances are:

• rather small as they have MWs below 500 g/mol; most of chemicals in the market are <500 g/mol;
• are bioavailable as they have logP values between -5 and 5. The former indicates high water solubility, the latter high octanol solubility;
• not highly insoluble, as the majority of substances have a solubility higher than 10^-2^ mol/L;
• and mostly non-ionisable substances, as only 25 substances have pKa values below 7.0. The software could only provide pKa predictions for 93 substances.

### 3.3 Chemical space

**Fig. 5:**
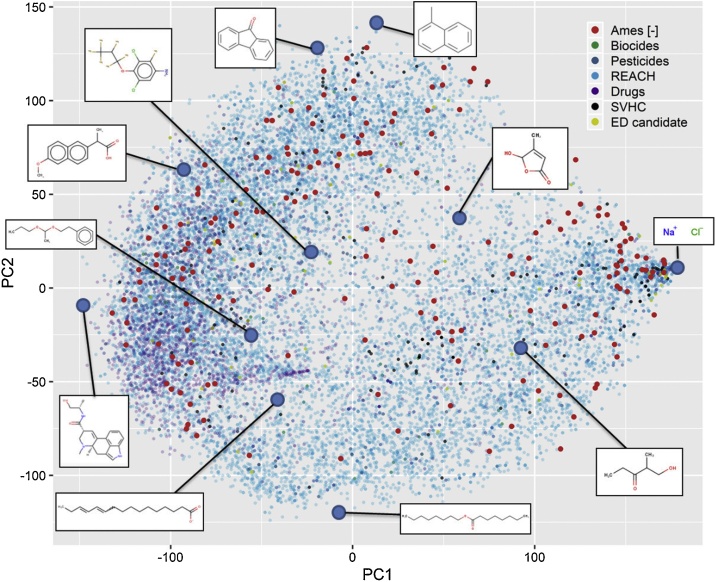
**Chemical space of the Ames-negative substances.** Chemical space of substances found in the Ames-negative database (207, red dots), and in the lists of REACH registered substances (15600), drugs (2388), pesticides (317), biocides (127), substances of very high concern (SVHCs) (199), and endocrine disruptor candidates (75) [[21], [22], [23], [24], [25], [26]]. The number of chemicals in each dataset corresponds to the number of substances for which a structure could be retrieved and is shown in brackets. Transition metal salts (e.g. Ti, V, Fe), natural products, and unknown, variable composition, or complex reaction products and biological materials (UVCBs) are not included. Axes and positions of the substances correspond to the first two principal components of the similarity matrix of the substances built using the RDKit [28] atomic pairs fingerprints. A total of 18904 substances are shown in the chemical space.

To describe the substances in the database in more detail, the chemical space covered by the substances was compared with that of other databases. Fig. 5 is a representation of the chemical space comparing different types of substances for which structures could be retrieved, i.e., REACH registered substances (n = 15600), drugs (n = 2388), pesticides (n = 317), biocides (n = 127), substances of very high concern (SVHC) (n = 199,) and endocrine disruptor candidates (n = 75) [[22], [23], [24], [25], [26], [27]], and the Ames-negative substances in the database (n = 207). For this reason, Gum arabic, Polysorbate 80, SD-0203 and SD-2007 were excluded from the analysis. In this type of plot substances are placed in an arbitrary coordinate system driven by the structural similarity to the other substances, e.g., the first two components of the principal component analysis of the similarity matrix. The dots that are found further apart correspond to substances that are structurally different. The structure of each chemical is represented by a fingerprint that is built from the pairs of atoms present in each molecule, the distance between them and the type of bonds. These types of fingerprints are named as “atomic pairs” fingerprints, and were calculated with the RDKit software [28]. The differences between chemicals is better observed in a 3D plot of the first three principal components, but the 2D plot of Fig. 5 is sufficient to capture the structural diversity of the substances in the database. For instance, the furthest right substance corresponds to Sodium chloride, the furthest left to Ergonovine, the top one to 1-Methylnaphthalene, and the bottom one to Octyl octanoate. Structures of given chemicals in the chemical space are shown for reference. Ames-negative substances are indicated as red dots. The fact that Ames-negative substances cover a vast area of the displayed space indicates that the Ames-negative database contains a structurally diverse set of substances. Some areas of the chemical space that are not well covered by Ames-negatives are the top left and bottom central area, which correspond to surfactants and adhesives (bottom central), and anti-inflammatory drugs, coatings and plasticisers (top left).

**Fig. 6:**
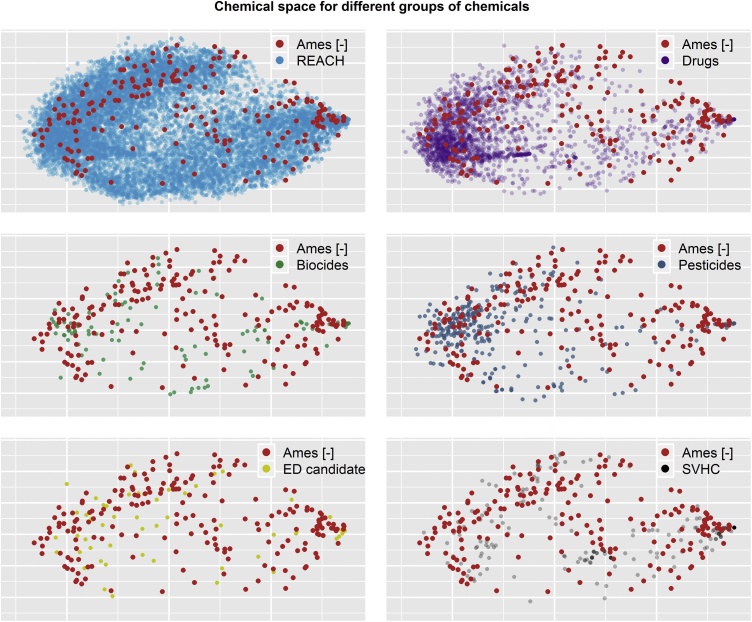
**Chemical space of the Ames-negative substances for different groups of chemicals.** Chemical space of substances found in the lists of REACH registered substances, drugs, pesticides, biocides, substances of very high concern (SVHC), and endocrine disruptor candidates. The superposition of Ames-negative substances with each of the groups is shown (i.e., red dots). Axes and positions of the substances correspond to the first two principal components of the similarity matrix of the substances built using the RDKit [28] atomic pairs fingerprints. A total of 18904 substances are shown in the chemical space. See caption of Fig. 5 for more information on the sources of the data.

Fig. 6 shows how the Ames-negative substances in the database compare to the different groups of substances included in Fig. 5. Fig. 5 showed that Ames-negative substances are rather well distributed in chemical space, with the exception that they are absent from the top left and bottom central part of the plot. In Fig. 6 this statement still holds, but only for REACH chemicals and Drugs. The other groups of chemicals, which are also less common, show fewer chemicals in those regions too. Therefore, the overlap of the Ames-negatives with these other groups (e.g., Biocides, Pesticides, etc.) seems to be higher. There is a region at the top middle-right where Ames-negative substances appear, and where biocides, pesticides, and SVHCs are not present (Fig. 6).

## 4 Preliminary analysis of the database

### 4.1 Testing patterns of the Ames-negative substances

There are a number of approaches that can be used to analyse the information contained in the database. Here, we present a preliminary synopsis regarding the distribution of those *in vitro* and *in vivo* genotoxicity tests with relevant results for the different substances in the database (Supplementary Table 1).

**Fig. 7:**
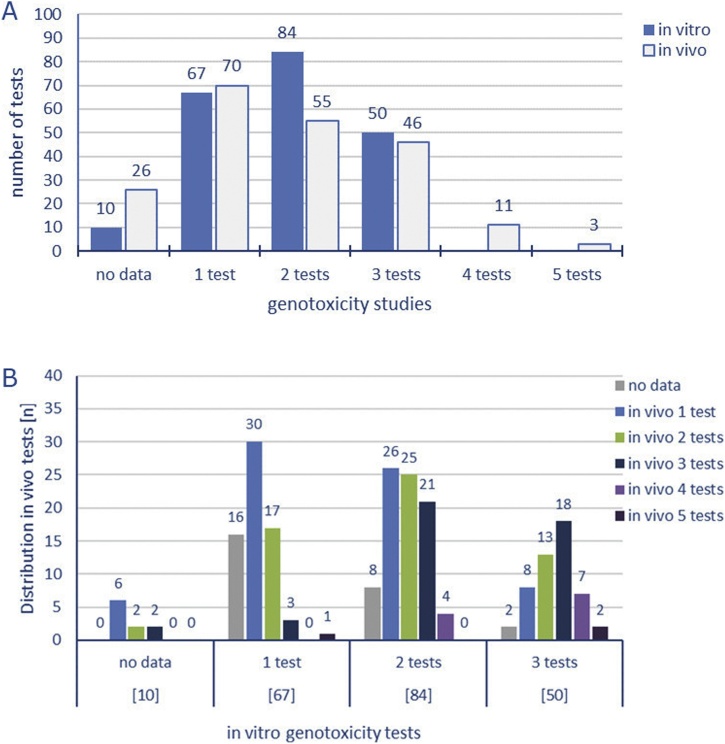
**Distribution of genotoxicity test results for Ames-negative substances.** A) Number of Ames-negatives tested in one, two or three *in vitro* or *in vivo* genotoxicity studies. B) Number of *in vivo* genotoxicity test results of Ames-negative substances that were either not tested, or tested in one, two or three *in vitro* genotoxicity studies. Only substances with valid genotoxicity test results, irrespective of their carcinogenicity test results, were considered. Inconclusive results were excluded.

As reported in Fig. 7A, approximately 32% (67/211) of the Ames-negative substances in the database have been tested in at least one *in vitro* mammalian cell test, approximately 40% (84/211) have been tested in two tests, mainly MLA and CA assay, and 24% (50/211) tested in all three mammalian cell tests, i.e., MCGM (*Tk+/−* or *Hprt*), MN and CA.

The number of Ames-negative substances that have been tested in one, two or three *in vivo* studies were 33%, 26% and 22%, respectively. The substances tested in one *in vitro* mammalian cell test (Fig. 7B), which have also been tested in one *in vivo* study (45%, 30/67), were mostly tested with the micronucleus assay (see also Fig. 2). The number of *in vivo* follow-up tests for each substance increases with the number of mammalian cell tests performed *in vitro* (Fig. 7B). Thus, the 40% (84/211) of Ames-negative substances tested in two *in vitro* tests have been equally tested in one, two or three *in vivo* studies. Also, most of the substances tested in three *in vitro* tests have also been tested in three *in vivo* tests. Only 11 of those had also been tested in four different *in vivo* test studies: Benzoin, Caprolactam, Di(2-ethylhexyl)phthalate, Dimethylformamide, Ethyl acrylate, Methyl tert-butyl ether, Methylphenidate hydrochloride, Nitrobenzene, Sodium arsenite, Vanadium Pentoxide (Divanadium pentoxide), and Wyeth 14,643 (4-Chloro-6-(2,3-xylidino)-2-pyrimidinylthio acetic acid). Less than 2% of all substances within the database (i.e., 3 out of 211) have been tested in five different *in vivo* studies; namely Acetaminophen, Acrylamide and Aroclor 1254.

### 4.2 Characterisation of the organic functional groups present in the Ames-negative database

The Ames-negative substances were further described using an analysis of organic functional group (OFG) frequency. The OECD QSAR Toolbox profiler named “Organic Functional Groups (Norbert Heider)” v2.0 [29] was used to categorise the OFGs in the substances of the Ames-negative database. This profiler analyses the chemical structures by searching for the presence of one or more of the 204 pre-coded OFG (e.g., ether, phenol, carbonic acid diester, nitrate). The list of possible OFG, and definition rules can be found at http://merian.pch.univie.ac.at/∼nhaider/cheminf/fgtable.pdf. A substance can have more than one OFG; a total of 335 OFGs were found in non-carcinogens and 502 in carcinogens. More specifically, 50% more OFGs were found in carcinogens than in non-carcinogens; there are 68% more carcinogenic substances in the database.

**Fig. 8:**
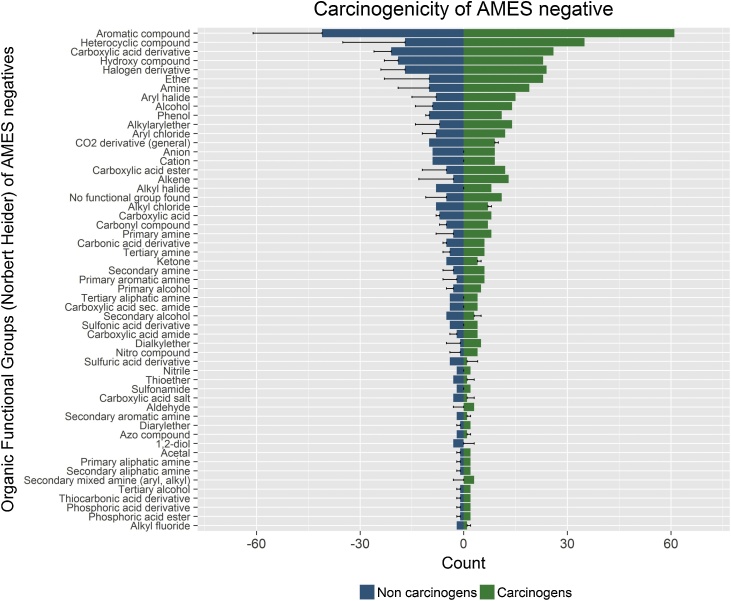
**The distribution of OFCs (Organic Functional Groups) for the Ames-negative substances in the database.** Bar plots showing the characteristics of the substances in the Ames-negative dataset as described by the number of fragments identified using the “Organic Functional Groups (Norbert Heider)” profiler (OECD QSAR Toolbox 4.1)[11,29]. For readability, only fragments found in more than 3 substances are shown. For each functional group, the error bars indicate differences in the numbers of rodent carcinogens and non-carcinogens.

If the OFGs had no role at all in the carcinogenicity of substances in the database, one would expect a similar prevalence of OFGs for carcinogens and non-carcinogens. On the other hand, a significant difference in the prevalence of a specific OFG in one of the subgroups (i.e., carcinogens vs non-carcinogens) may indicate a possible mechanism that could be scrutinised by further analysis. Fig. 8 shows the OFGs for Ames-negative database substances, and their frequency for carcinogens (green bars) and non-carcinogens (blue bars), respectively

The most common OFG found in the Ames-negative database was aromatic compound, which corresponds to the presence of any conjugated ring system. This was found for a total of 102 substances; aromatic compounds are 48% more prevalent in carcinogens than in non-carcinogens. Heterocyclic substances, which include those with a cyclic structure containing at least one non-carbon atom, was the second most common fragment in the database. It was found for 52 substances, with 105% more heterocyclic fragments in carcinogens compared to non-carcinogens. Ethers and amines followed the same trend; occurring more frequently in carcinogens than non-carcinogens.

The rest of the OFGs, which include aliphatic amines and alkenes, among others, are likely too rare for meaningful analysis. Still, it is perhaps interesting to point out the higher frequency of alkenes in carcinogens (13 fragments vs 3). Most of the alkene-containing substances in the database were found in aromatic substances too, and therefore the distribution of alkenes between Ames negative carcinogens and non-carcinogens would not be expected to be very different from that of aromatic substances. However, non-aromatic alkenes (e.g., d-limonene, isobutene) were found to occur more frequently in carcinogens (5 vs 0).

## 5 Discussion

The construction of a consolidated database of genotoxicity and carcinogenicity results for >700 Ames-positive chemicals, which was developed [7,8] as a follow-up activity of an EURL ECVAM workshop (2013), led to the public release of a powerful data resource intended to aid in the interpretation of *in vitro* genotoxicity results in general, likewise implementing the EURL ECVAM strategy on genotoxicity testing [3]. As such, the EURL ECVAM Consolidated Genotoxicity and Carcinogenicity Database of Ames-positives has, over recent years, become a reference for a number of regulatory activities in the area of genotoxicity testing across different product-type sectors [30]. Among those, the analysis of this database contributed to the Addendum of the European Scientific Committee on Consumer Safety (SCCS)'s Notes of Guidance for testing cosmetic ingredients and their safety evaluation [31]. The European Food Safety Authority (EFSA) also relied on the analysis of the EURL ECVAM database data to provide advice on the suitability of the *in vivo* unscheduled DNA synthesis (UDSviv) assay to follow-up positive results in *in vitro* gene mutation tests of substances used in food and feed [32].

The availability of a robust set of Ames positive data has served as a reference for exploratory projects, development of testing strategies, evaluation and validation of several new genotoxicity tests, including the ongoing evaluation of the miniaturised versions of the Ames test being carried out by the OECD [33]. Recently, the database has been also used as a data source to develop an animal-free strategy for the prioritization of substances of genotoxic concern, specifically food contact materials [34]. Moreover, the Health and Environmental Sciences Institute’s (HESI) Genetic Toxicology Technical Committee used part of the information stored in the database to provide more detailed advice on which *in vivo* test to choose to follow-up on *in vitro* positive results [35], and to inform case studies to illustrate the approach for a next-generation testing strategy for assessment of genomic damage [36].

Based on the above, and the regulatory implications of Ames test results, it was considered beneficial to extend the aforementioned database to include Ames-negative substances. With this objective, a compilation of Ames-negative data was initiated, leading to a robust and highly curated database of 211 Ames-negative substances. The complementary data included in the database, corresponding to other genotoxicity endpoints *in vitro* and *in vivo*, as well as carcinogenicity results, has resulted in a database with approximately 6000 test results. This Ames-negative database is user-friendly and freely-accessible (Supplementary Table 1).

The database includes substances across different product-type sectors; the most abundant are industrial chemicals, pesticides and pharmaceuticals. Biocides, food additives, flavouring agents and natural products are equally represented; only 2% are cosmetic ingredients (Fig. 3). The chemical space covered by the Ames-negative substances, which is displayed in Fig. 5, Fig. 6, showed that the Ames-negative substances are rather well distributed in the chemical space covered by REACH registered substances, drugs, pesticides, biocides, substances of very high concern [SVHC], and endocrine disruptor candidates. The exceptions were surfactants and adhesives, anti-inflammatory drugs, coatings and plasticisers that are not well represented amongst the Ames-negative substances in this database. For the most represented product types, the analysis of genotoxicity test results can permit an evaluation of the genotoxicity strategy currently in place, and the possibility to improve the testing recommendations for substances within the specific substance categories.

It is also conceivable that the database could serve as a platform for structural and/or functional characterization of specific groups of substances with or without carcinogenic and/or *in vivo* genotoxic activity. For example, by analysing the presence of certain types of OFGs in Ames-negative carcinogens and non-carcinogens [11], it may be possible to identify structural determinants of carcinogenic potential. In the Ames-negative database the number of OFGs found in carcinogens was 50% higher than that found in non-carcinogens, and the most common OFG was aromatic compound. The prevalence of the aromatic compound OFG, which was 48% higher in carcinogens than non-carcinogens, does not necessarily indicate any relationship between aromatic compound OFG and Ames-negative carcinogenicity since it is almost identical to the prevalence of the OFG in the database overall. In contrast, heterocyclic fragments, ethers, amines and alkenes seem to be considerably more common in carcinogens than non-carcinogens; possibly indicating the relevance of these OFGs in the carcinogenicity of Ames-negative substances, although more substances would be needed to confirm such a functional relationship.

In light of the limited data for some OFGs, a more in-depth analysis would be required in order to scrutinise empirical relationships between carcinogenicity and the relative frequency of the various OFGs. Such an analysis is particularly important for substances that act via a physical effect, and whose carcinogenic mechanism is not related to their OFGs (see highlighted entries in Supplementary Table 1).

The information contained in the Ames-negative database can also be used in analyses investigating the carcinogenic MOA of non-genotoxic substances, e.g., those that act via physical damage or hormonal alterations. Such an analysis would also be used to scrutinise *in vivo* genotoxins, highlighting those substances suspected of inducing genotoxic effects via alterations of homeostasis manifested as hypothermia, erythropoiesis, methaemoglobinaemia, etc. In both cases, negative Ames results would be expected, and therefore any correlation/predictivity analysis would be more meaningful if such substances were excluded.

Furthermore, it is expected that the information described herein can be used to support ongoing projects aimed at determining the mechanisms underlying the genotoxicity and/or carcinogenicity of specific classes of substances and/or substances with specific OFGs, thus linking structural alerts and/or mechanistic domains to the effects indicated by the results of *in vitro* and *in vivo* genotoxicity studies [37,38]. Indeed, the information on genotoxicity and carcinogenicity of Ames-positive chemicals has been already included in the OECD QSAR Toolbox [11] and the new set of curated data will likely enrich and advance the use of this tool. In addition, the curated data can be used to support the development of novel tools (e.g., *in silico* tools that more effectively predict Ames test results [39], as well as novel genotoxicity assessment approaches. The latter could include integrated testing strategies that consider the relationships between chemical properties, genotoxicity results and carcinogenicity results, thus providing more effective and accurate evaluations of carcinogenicity hazard [40].

Overall, it is reasonable to assert that in-depth analysis of the Ames-negative database could be used to address a host of pertinent questions. For example:

• For Ames negative carcinogens and non-carcinogens, what are the frequencies of positive and negative results in other *in vitro* and *in vivo* genotoxicity tests?
• Can other genotoxicity tests/endpoints be employed to effectively predict the carcinogenic activity of Ames-negative substances? And, if so, which *in vitro* and/or *in vivo* genotoxicity tests and/or which combinations of tests?
• Which combinations of *in vitro* tests effectively predict *in vivo* genotoxic activity?

Answers to such questions may provide information that can be employed to identify reference substances that more effectively assess the performance of new and/or improved genotoxicity tests [17,41]. Answers might also provide information that can be used to identify, for example, those substances that act via an indirect mode of action (e.g., aneugens, topoisomerase inhibitors, metabolic poisons).

Lastly, answers to the aforementioned questions will very likely facilitate determination of those circumstances (e.g., specific classes of compounds and/or specific chemical properties) where new targets of genomic damage might be identified and eventually be used to provide additional mechanistic evidence for assessment of genotoxic and/or carcinogenic hazard [[42], [43], [44]]. In-depth analysis of the database is on-going; the results of these analyses will be presented in a forthcoming manuscript.

## 6 Conclusions

After consideration regarding the impact and utility of the EURL ECVAM Consolidated Genotoxicity and Carcinogenicity Database for regulatory genotoxicity assessment, we have herein extended the database to include Ames-negative substances. The update is considered an important step towards improvement of genotoxicity hazard identification. More specifically, the new database, which contains rigorously curated information, will permit improved interpretation of genotoxicity test results, improved ability to evaluate the efficacy and accuracy of current testing regimes, and indeed, improved ability to accurately and effectively identify genotoxic substances that are active *in vivo*.

## Funding sources

This research did not receive any specific grant from funding agencies in the public, commercial, or not-for-profit sectors. D.K. received financial support to help compile and revise the entries in the database under EC-JRC CT-EXP2013D136810-101,-102.

## Disclaimer

This document represents the consensus of the authors’ views expressed as individual scientists and does not necessarily represent the policies and procedures of their respective institutions.

## Appendix A Supplementary data

Table S1 XXXXX

## Footnotes

Appendix A Supplementary material related to this article can be found, in the online version, at doi:https://doi.org/10.1016/j.mrgentox.2020.503199.
