# Corrigendum to “EURL ECVAM genotoxicity and carcinogenicity database of substances eliciting negative results in the Ames test: Construction of the database” [Mutat. Res. 854–855 June–July (2020) 503199]

**DOI:** 10.1016/j.mrgentox.2020.503274

**Published:** 2020

**Authors:** Federica Madia, David Kirkland, Takeshi Morita, Paul White, David Asturiol, Raffaella Corvi

**Affiliations:** a European Commission Joint Research Centre, Ispra, Italy; b Kirkland Consulting, Tadcaster, UK; c Chemical Management Center, National Institute of Technology and Evaluation (NITE), Nishihara, Shibuya-ku, Tokyo, Japan; d Environmental Health Science and Research Bureau, Health Canada, Ottawa, Canada

In online Appendix A, Supplementary data, the overall call for Benzoin, in vivo UDS, should be [-].

In the table:

Column CC, row 31: change “[+] in rat hepatocytes#Glauert et al. 1985” to “[+] in vitro rat hepatocytes#Glauert et al. 1985” .

Column CD, row 31: change “[+]” to “[-]”.

The authors apologise for any inconvenience caused.

